# Kawasaki Disease presenting with intussusception: a case report

**DOI:** 10.1186/1824-7288-36-7

**Published:** 2010-01-19

**Authors:** Rumana N Hussain, Gary Ruiz

**Affiliations:** 1 Northampton General Hospital, Northampton, UK; 2 King's College Hospital, London, UK

## Abstract

A 3 yr old boy presented with abdominal pain, fever and red jelly stools. Intussusception was diagnosed and effectively reduced with air insufflation. However, despite an improvement in his clinical condition, the child remained febrile and miserable; 5 days later he developed characteristic signs of Kawasaki disease and was treated with intravenous immunoglobulin and high dose aspirin with good results. Intussusception prior to the typical features of Kawasaki disease has not been described previously in the English literature. This case illustrates a novel presentation of Kawasaki disease.

## Case

A 3 yr old Caucasian boy was referred from the accident and emergency department to the paediatric surgical team with a history of acute onset abdominal pain. The pain was central, and associated with fever, non bilious vomiting and the passage of red, jelly-like stools. There was no history of weight loss, jaundice or foreign travel; there was no contact history and no recent change in dietary habits. Other than a mild upper respiratory tract infection, the child was previously fit and well with no relevant medical history. On examination, the child was well nourished; he was miserable and lethargic with fever (38.1°C) and tachycardia (heart rate 120/min). He was warm and well perfused with a capillary refill time of <2 seconds both centrally and peripherally. His abdomen was tender, distended and resonant; bowel sounds were hyperactive and a small, tender, rubbery mass was palpable centrally. An ultrasound scan was undertaken and confirmed the presence of an intussusception. The child underwent a contrast enema; control films showed a filling defect in the ascending colon at the location of the colo-colic intussusception. During a subsequent air enema, air insufflation to a pressure of 120 mmHg was sufficient to reduce the intussusception adequately. The child was kept in hospital overnight for observation.

By the following day, the boy's condition had improved with resolution of his abdominal pain; he was able to eat and drink and interact with his parents and staff. However he continued to have a remittent fever to 38.5°C and he remained lethargic and miserable. Subsequently, he developed a diffuse morbilliform rash over the trunk and limbs and cervical lymphadenopathy was noted with an associated congested throat and enlarged tonsils, which was thought to be viral in origin. Blood tests revealed a C-reactive protein 61 mg/L, normal blood count, serum electrolytes, liver function tests and chest X-ray; Ebstein Barr viral serology, blood cultures and urine cultures were negative. Five days after initial presentation, he developed conjunctival oedema and oedema of the hands and feet with desquamation of the finger tips and cracking of the lips; there was no evidence of a strawberry tongue. Kawasaki Disease was diagnosed. The child was treated with a dose of intravenous immunoglobulins (2 g/kg) and initiated on a therapy of high dose aspirin (25 mg/kg QDS). He made a good recovery and was discharged 4 days later. The aspirin was altered to a lower dose (5 mg/kg OD) after 2 weeks following an initial normal echocardiogram. Echocardiography was repeated at 8 weeks and showed no coronary artery abnormality. The aspirin was subsequently stopped.

## Discussion

Intussusception is the commonest cause of intestinal obstruction in infants, and occurs with an incidence of approximately 66/100,000 in the UK [1]. Lead points may include polyps, lymph nodes, diverticulae. However in most cases the cause is unknown and is thought to be due to enlarged Peyers patches; enlarged intestinal lymph nodes have been described in those requiring surgical intervention[2,3]. Intestinal lymphadenopathy can occur for many reasons such as Crohns disease, tumours, gastrointestinal infections or drug reactions; however enlarged Peyer's patches occur most often in young children as a reaction following viral upper respiratory tract or gastrointestinal infection. Intussusception is therefore unsurprisingly more common in the spring and autumn seasons[4]. This theory is further strengthened by reports of an increasing incidence of intussusception following the introduction of the rotavirus vaccination in the US[5].

Kawasaki Disease is similarly postulated to be triggered by an initial infective insult, with a similar preceding history of a viral upper respiratory or gastrointestinal infection, and similar seasonal pattern of disease[6]. On presentation, a proportion of patients (approximately 40%) exhibit cervical lymphadenopathy; however whether children have concurrent lymphadenopathy elsewhere is unknown. Development of intestinal mucosal lymphadenopathy due to the same trigger viral pathogen may explain the process by which intussusception occurs in these children.

Gastrointestinal symptoms are common in Kawasaki disease; abdominal pain, nausea and diarrhoea are seen in up to one third of cases, and these are therefore often regarded as symptoms of Kawasaki disease rather than of potential intra abdominal complications. Symptoms often resolve on treatment with intravenous immunoglobulins. The development of an acute surgical abdomen is much less common but well documented. The most well recognised intra abdominal pathology is of gallbladder hydrops, occurring in up to 15% of cases of Kawasaki disease; there have also been reports of intestinal obstruction and pseudo obstruction, or more rarely pancreatitis, appendicitis, ischaemic colitis and intussusception[7,8].

There have been only three previous reports of intussusception associated with Kawasaki disease [9-11]. There are 2 cases, a 3 year old girl [9] and a 4 month old girl[10], initially presenting with symptoms of Kawasaki disease, diagnosed and treated as such, and subsequently developing abdominal symptoms of episodic pain, vomiting, distension and bloody stool during their period of hospitalization. A diagnosis of intussusception was made in each case (ileocolic/ileocaecal) with barium studies, and the intussusceptum reduced without complication by enema. The younger girl of 3 months of age developed coronary artery aneurysms, evident on echocardiography. There has been just one case, a 3 month old boy [10], initially presenting with abdominal symptoms with a resulting primary diagnosis of a colo-colic intussusception, and soon after developed symptoms characteristic of Kawasaki disease and was thereby subsequently treated with immunoglobulins. This child also developed coronary artery aneurysms.

This is the first report in the English literature of a child presenting with an intussusception *with no initial symptoms of Kawasaki disease*. The two conditions were highly likely to have been associated in this case. It is important to consider underlying causes of intussusception in this older age group of children, and consider Kawasaki disease in those febrile on presentation. We wish to highlight this novel presentation of Kawasaki disease.

## Abbreviations

CRP: C-reactive protein.

## Consent

Written informed consent was obtained from the patient's parents for publication of this case report. A copy of the written consent is available for review by the Editor-in-Chief of this journal.

## Competing interests

The authors declare that they have no competing interests.

## Authors' contributions

All authors read and approved the final manuscript

## References

[B1] Gay N Ramsay M Waight P Rotavirus vaccination and intussusception The Lancet 1999 354 9182 956 10.1016/S0140-6736(05)75710-X 10489989

[B2] Nissan S Levy E Intussusception in infancy caused by hypertrophic Peyer's patches Surgery 1966 59 6 1108 11 5937957

[B3] Sarason EL Proir JT Prowda RL Recurrent Intussusception associated with hypertrophy of Peyer's patches N Eng J Med 1955 253 21 905 8 10.1056/NEJM195511242532104 13272813

[B4] Eschel G Barr J Heyman E Tauber T Klin B Vinograd I Starinsky R Lahat E Intussusception: A 9-year Survey (1986-1995) J Pediatric Gastroenterology and Nutrition 1997 24 3 253 256 10.1097/00005176-199703000-00004 9138168

[B5] Murphy TV Gargiullo PM Massoudi MS Nelson DB Jumaan AO Okoro CA Zanardi LR Setia S Fair E LeBaron CW Wharton M Livengood JR Rotavirus Intussusception Investigation Team. Intussusception among infants given an oral rotavirus vaccine N Engl J Med 2001 344 564 72 10.1056/NEJM200102223440804 11207352

[B6] Porcalla AR Sable CA Patel KM Martin GR Singh N The Epidemiology of Kawasaki Disease in an Urban Hospital: Does African American Race Protect Against Coronary Artery Aneurysms? Pediatr Cardiol 2005 26 775 781 10.1007/s00246-005-0916-5 16421770

[B7] Yaniv L Jaffe M Shaoul R The surgical manifestations of the intestinal tract in Kawasaki disease (2005) Journal of Pediatric Surgery 2005 40 9 e1 e4 10.1016/j.jpedsurg.2005.05.063 16150324

[B8] Zulian F Falcini F Zancan L Martini G Secchieri S Luzzatto C Zacchello F Acute surgical abdomen as presenting manifestation of Kawasaki disease J Pediatr 2003 142 6 731 5 10.1067/mpd.2003.232 12838207

[B9] Caty MG Stylianos S Lillehei CW Ileocolic intussusception in Kawasaki's disease Pediatric Surgery International 2004 9 8 590 591

[B10] Huang YC Lin TY Su WJ Unusual manifestations in children with Kawasaki disease J Formos Med Assoc 1997 96 6 451 6 9216170

[B11] Tori Tori C Bazan Zender C Vargas Galgani M Enfermedad de Kawasaki: A propósito de un caso atípico y con intususcepción Rev Med Hered, ene 2001 12 1 37 41

